# Tumor-recruited M2 macrophages promote gastric and breast cancer metastasis via M2 macrophage-secreted CHI3L1 protein

**DOI:** 10.1186/s13045-017-0408-0

**Published:** 2017-02-01

**Authors:** Yulei Chen, Siyuan Zhang, Qizhi Wang, Xiaobo Zhang

**Affiliations:** 1 College of Life Sciences and Laboratory for Marine Biology and Biotechnology of Qingdao National Laboratory for Marine Science and Technology, Zhejiang University, Hangzhou, 310058 People’s Republic of China; 2 Department of Gastroenterology, The First Affiliated Hospital of Bengbu Medical College, Bengbu, 233030 People’s Republic of China

**Keywords:** AP-1, Cancer metastasis, CHI3L1, IL-13Rα2, M2 macrophage

## Abstract

**Background:**

The macrophage, one of the several key immune cell types, is believed to be involved in tumorigenesis. However, the mechanism of macrophages promoting tumor progression is largely unknown.

**Methods:**

The differentially secreted proteins of M1 and M2 macrophages were analyzed by mass spectrometry. We performed GST pull-down assay for the identification of cell-membrane receptors that interact with chitinase 3-like protein 1 (CHI3L1) protein. The mouse model was used to validate the function of CHI3L1 in cancer metastasis in vivo. Protein phosphorylation and gene expression were performed to study the signaling pathway activation of cancer cells after CHI3L1 treatment.

**Results:**

M2 macrophage-secreted CHI3L1 promoted the metastasis of gastric and breast cancer cells in vitro and in vivo. The CHI3L1 protein functioned by interacting with interleukin-13 receptor α2 chain (IL-13Rα2) molecules on the plasma membranes of cancer cells. Activation of IL-13Rα2 by CHI3L1 triggered the activation of the mitogen-activated protein kinase signaling pathway, leading to the upregulated expression of matrix metalloproteinase genes, which promoted tumor metastasis. The results of this study indicated that the level of CHI3L1 protein in the sera of patients with gastric or breast cancer was significantly elevated compared with those of healthy donors.

**Conclusions:**

Our study revealed a novel aspect of macrophages with respect to cancer metastasis and showed that CHI3L1 could be a marker of metastatic gastric and breast cancer in patients.

**Electronic supplementary material:**

The online version of this article (doi:10.1186/s13045-017-0408-0) contains supplementary material, which is available to authorized users.

## Background

Cell migration is an essential process for the development and maintenance of multicellular organisms and is defined as the orchestrated movement of cells in a particular direction to a specific location [1]. To migrate, a cell must modify its shape to be able to interact with the surrounding tissue structures. Therefore, the extracellular matrix (ECM) serves as a substrate as well as a barrier for an advancing cell body [2]. Cell migration is an integrated, multistep process that contributes to tissue repair and regeneration, orchestrates embryonic morphogenesis and drives disease progression. During cell migration, the moving cell first becomes polarized and elongated. A pseudopod is then formed, which attaches to the ECM substrate. Subsequently, regions of the leading edge contract, thereby generating a traction force that leads to the gradual forward gliding of the cell body and its trailing edge. Metastasis, which involves the migration of cancer cells from the primary tumor to a distant organ or tissue, is the most frequent cause of death for patients with cancer. Cancer cells utilize cell migration mechanisms that are similar to those exhibited by non-neoplastic cells during physiological processes [2]. The steps of metastasis include tumor cell adhesion to and invasion of basement membranes and the surrounding tissue, intravasation into blood vessels, survival in the bloodstream, extravasation, and growth at different organ sites [2]. Only a small proportion of tumor cells invade and disseminate. However, the molecular mechanisms underlying metastasis are still poorly understood because of their apparent complexity.

Tumor progression is controlled by crosstalk between cancer cells and other cell types within the tumor microenvironment. The tumor microenvironment includes proliferating tumor cells, blood vessels, the tumor stroma, inflammatory cells, and a variety of associated tissue-type cells. This unique environment forms over the course of tumor progression due to the interactions of tumor cells with the host. This microenvironment is shaped and dominated by the tumor, which orchestrates molecular and cellular events occurring in the surrounding tissues. Although various immune effector cells are recruited to the tumor site, their anti-tumor functions are downregulated, largely in response to tumor-derived signals. Immune effector cells in the tumor microenvironment not only fail to perform anti-tumor functions but are co-opted to promote tumor growth [3]. Macrophages are a key component of the tumor microenvironment that act as facilitators of tumor cell migration and invasion, matrix degradation, and angiogenesis. The density of macrophages in the tumor microenvironment has been found to be a prognostic marker of poor outcome for a variety of carcinomas. Evidence from clinical and experimental studies has indicated that tumor-associated macrophages (TAMs) promote solid-tumor metastasis by releasing a variety of cytokines, including chemokines, inflammatory factors, and growth factors [4–6]. For example, macrophages can promote angiogenesis by secreting IL-1, VEGF, and MMP-2 [7]. Growth factors and proteases produced by macrophages can initiate tumorigenesis and enhance tumor progression [8]. However, many cytokines that are produced by cancer cells themselves also contribute to carcinogenesis [9]. Elevated cytokine expression may be a diagnostic cancer marker. Currently, TAM cytokine profiles are largely unknown. It is unclear whether there are some exclusively TAM-derived cytokines that are functionally essential to tumor progression.

There are different types of macrophages, such as M1 and M2 macrophages [10]. Phagocytosis mediated by macrophages (M1 macrophages) is essential to the immune response of animals, as revealed in our previous studies [11–14]. M2 macrophages can suppress inflammatory responses as well as promote angiogenesis and tissue remodeling and repair [10]. Our previous studies indicated that inhibition of microRNA-100 triggered apoptosis of breast and cancer cells by inhibiting ubiquitination-mediated p53 degradation [15, 16], revealing a vital role of microRNAs in tumor progression. To further explore the role of macrophages in tumor progression, the proteins secreted by macrophages were characterized in this study. The results indicated that M2 macrophages are recruited by tumor cells. The M2-secreted chitinase 3-like protein 1 (CHI3L1) promoted the metastasis of gastric and breast cancer cells by triggering the mitogen-activated protein kinase (MAPK) signaling pathway. Therefore, our study revealed a novel macrophage-mediated mechanism that underlies cancer cell metastasis.

## Methods

### Identification of CHI3L1 secreted by M2 macrophages

M1 and M2 macrophages were cultured in serum-free medium for 24 h. Then, the culture supernatants were collected and were dialysed against 1 L of distilled water overnight at 4 °C. The secreted proteins were resolved on a 15% SDS-PAGE denaturing gel and were visualized using Coomassie Brilliant Blue. The protein band of interest was removed for mass spectrometry analysis.

### Identification of cell-membrane receptors that interact with CHI3L1 protein

GST (glutathione S transferase)-conjugated CHI3L1 protein was expressed in *E. coli* BL21 cells and was purified using standard protocols. Glutathione-Sepharose beads (GE Healthcare, Waukesha, WI, USA) coupled with either GST or with the GST-CHI3L1 purified protein were incubated with the solubilized membrane proteins for 1 h at 4 °C. The membrane proteins of the gastric and breast cancer cells were extracted using a ProteoExtract Native Membrane Protein Extraction kit (Calbiochem, San Diego, CA, USA) according to the manufacturer’s instructions. After rinsing the beads three times with washing buffer (50 mM HEPES-KOH, 150 mM NaCl, 1 mM MgCl_2_, 0.2% Triton-X-100, pH 7.2), the proteins bound to the beads were separated using 10% SDS-PAGE and were visualized using Coomassie Brilliant Blue R-250 staining. The differentially apparent proteins were excised from the gel and were identified using mass spectrometry.

### Assessment of breast cancer metastasis in vivo

The breast cancer metastasis assay was conducted in mice. All the experiments using animals were performed in accordance with a protocol approved by the Institutional Animal Care and Use Committee (IACUC). Female nude mice of between 5 and 6 weeks old were used in this study. Breast cancer cells (i.e., 2 × 10^5^ MDA-MB-231 cells or 8 × 10^5^ MDA-MB-435 cells) stably expressing the firefly luciferase reporter were mixed with 100 μl of PBS, and the mixture was intravenously injected into the mice. 3 days later, either recombinant CHI3L1 protein (rCHI3L1) or PBS (as the control) was injected into the mice via the tail vain at a dosage of 100 μg/kg of body weight. rCHI3L1 or PBS was injected twice a week over a 7-week (MDA-MB-231) or 11-week period (MDA-MB-435). For in vivo imaging, the mice were given the substrate D-luciferin by intraperitoneal injection at a dosage of 150 mg/kg in PBS, after which lung metastasis was quantified every 2 weeks by bioluminescence imaging using an IVIS Spectrum Imaging System (Perkin Elmer). Bioluminescence analysis was performed using Living Image software version 4.5 (Perkin Elmer). The solid tumors of mouse lungs were harvested at the end of the experimental period for further evaluation.

### Detection of CHI3L1 protein in the sera of healthy donors and metastatic cancer patients

Serum samples were obtained from patients in The First Affiliated Hospital of Bengbu Medical College, China. The samples were collected with the informed consent of the patients, and all related procedures were performed with the approval of the internal review and ethics boards of the indicated hospital. For the co-immunoprecipitation assay, the sera were centrifuged at 12,000 × *g* and 4 °C for 10 min. Then, the supernatants were diluted in EBC lysis buffer (50 mM Tris–HCl, 120 mM NaCl, and 2 mM PMSF). To remove the antibodies from the sera, the supernatants were incubated with Dynabeads® protein G (Invitrogen) with gentle rotation at 4 °C for 2 h. After centrifugation at 5,000 × *g* for 5 min, the supernatants were incubated with the anti-CHI3L1 IgG-conjugated Dynabeads® protein G with gentle rotation at 4 °C overnight. Subsequently, the mixture was washed twice using EBC lysis buffer and was analyzed by western blotting using the anti-CHI3L1 IgG.

### Statistical analysis

All biological experiments were repeated three times independently. Numerical data were analyzed using a one-way analysis of variance. The statistical significance between treatments was analyzed using Student’s *t* test.

## Results

### Tumor recruits M2 macrophages

**Fig 1:**
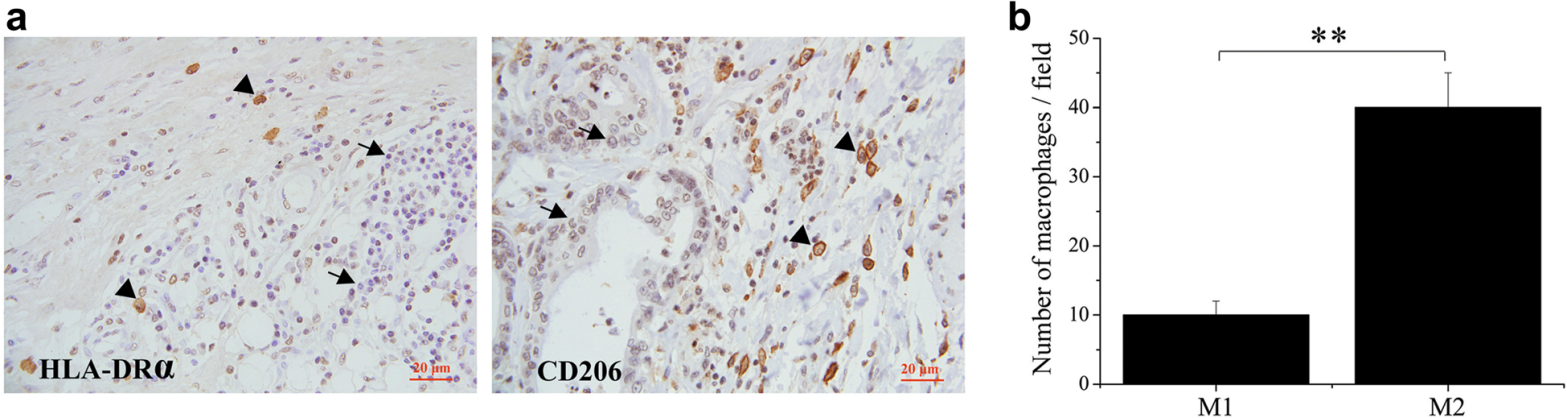
Tumor recruits M2 macrophages. **a** Immunohistochemical staining of HLA-DRα (an M1 macrophage marker) and CD206 (an M2 macrophage marker) in solid tumors from patients with gastric cancer. The tumor cells were stained with hematoxylin. The *arrows* indicate cancer cells, and the *triangles* indicate macrophages. *Scalebar*, 20 μm. **b** Numbers of M1 and M2 macrophages in tumor tissues

To characterize the types of macrophages that participate in tumorigenesis, solid tumors from patients with gastric cancer were immunohistochemically analyzed by staining for human leukocyte antigen-DRα (HLA-DRα, an M1 macrophage marker) and CD206 (an M2 macrophage marker). The results showed that more CD206-positive macrophages than HLA-DRα-positive macrophages were present in the cancerous tissues (Fig. 1a, b). These findings indicated that cancer cells recruited M2 macrophages (i.e., tumor-associated macrophages) into the tumor microenvironment.

### Peripheral blood monocytes-derived M2 macrophages promote cancer cell metastasis

**Fig 2:**
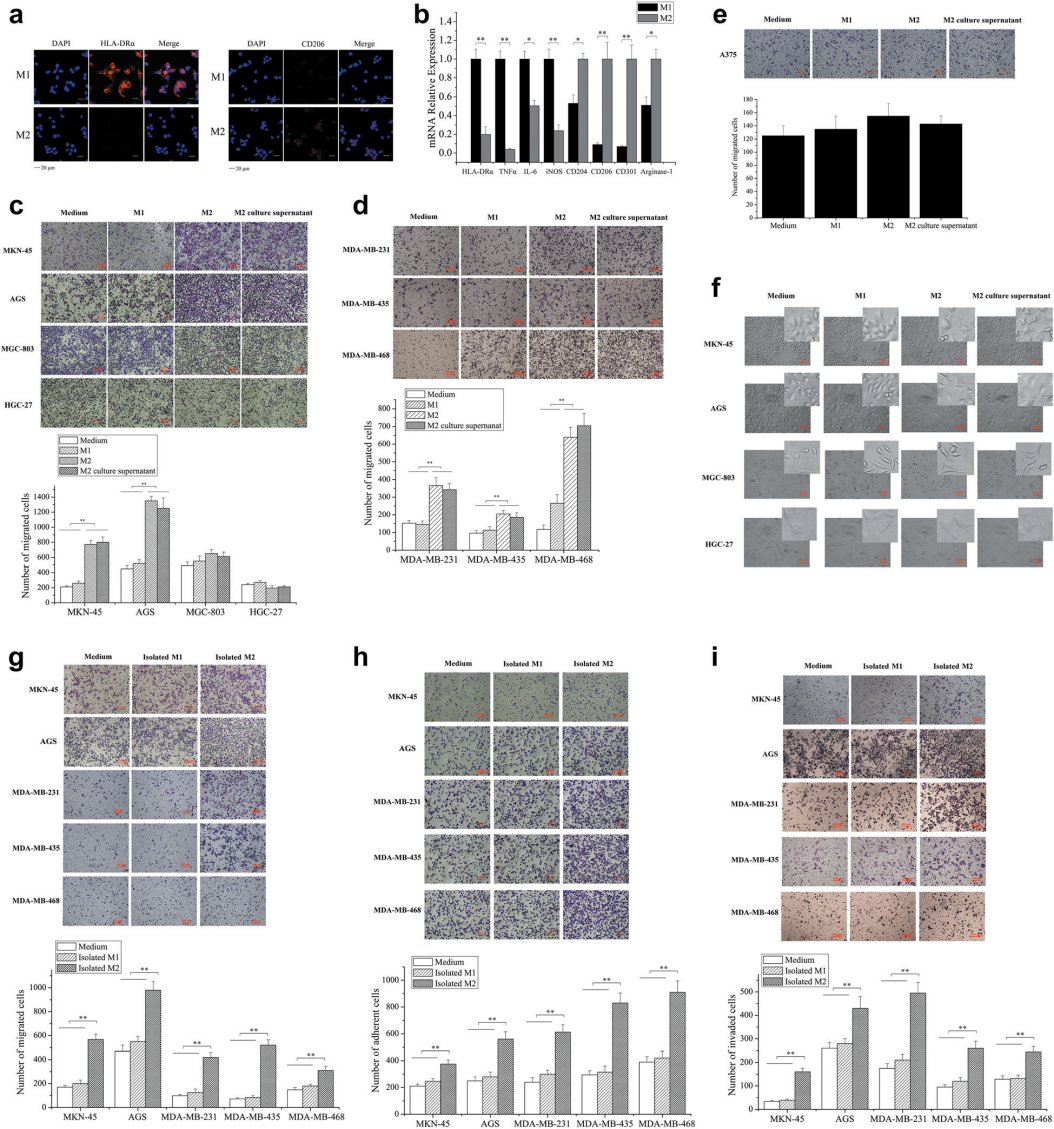
M2 macrophages promote cancer cell metastasis. **a** Confocal microscopy of immunostained macrophages. THP-1 monocytes were polarized into M1 or M2 macrophages. Macrophages were labeled with anti-HLA-DRα or anti-CD206. The nuclei were counterstained with DAPI. *Scalebar*, 20 μm. **b** Gene expression profiles of M1 and M2 macrophages. M1 and M2 macrophage gene expression levels were quantified using real-time PCR. The genes are shown on the horizontal axis. **c**, **d**, **e** Cancer cell migration characterized using Boyden chamber assays. Gastric cancer cells (MKN-45, AGS, MGC-803, and HGC-27), breast cancer cells (MDA-MB-231, MDA-MB-435, and MDA-MB-468), or melanoma cells (A375) were co-cultured with M1 macrophages (M1), M2 macrophages (M2), or M2 culture supernatant. The culture medium alone (medium) was included in the co-culture study as the control. After 12 h of co-culture, cell migration was evaluated. *Scalebar*, 100 μm. **f** The effect of co-culture on gastric cancer cell morphology. MKN-45, AGS, MGC-803, or HGC-27 gastric cancer cells were co-cultured with culture medium (medium), M1 macrophages (M1), M2 macrophages (M2), or M2 culture supernatant. 24 h later, the cancer cells were examined by phase-contrast microscopy. *Scalebar*, 50 μm. **g** Effects of peripheral blood monocytes-derived M2 macrophages on gastric and breast cancer cell migration. The peripheral blood monocytes of healthy humans were induced to differentiate into M1 macrophages using LPS or into M2 macrophages using IL-4. Isolated M1 and M2 cells were then co-cultured with cancer cells. Cancer cell migration was examined using a Boyden chamber assay. *Scalebar*, 100 μm. **h** The role of peripheral blood monocytes-derived M2 macrophages in gastric and breast cancer cell adhesion. Cancer cells were co-cultured with the isolated M1 or M2 macrophages and subjected to Boyden chamber assays. *Scalebar*, 100 μm. **i** The influence of peripheral blood monocytes-derived M2 macrophages on gastric and breast cancer cell invasion. Gastric or breast cancer cells were co-cultured with isolated M1 or M2 cells, and then the cancer cells were analyzed using the invasion assay. *Scalebar*, 100 μm. In all the panels, the significant differences are indicated by *asterisks* (*p < 0.05, **p < 0.01)

To explore the effects of M2 macrophages on cancer cell metastasis, THP-1 monocytes were polarized into M1 and M2 macrophages. After this polarization, the macrophages were co-cultured with tumor cells. Confocal microscopy demonstrated that monocytes could be polarized into M1 or M2 macrophages (Fig. 2a). The gene expression profiles that were determined revealed that the expression levels of M1-associated genes (i.e., HLA-DRα, TNFα, IL-6, and iNOS) were significantly upregulated in M1 macrophages, whereas those of the M2-associated genes, including CD204, CD206, CD301, and Arginase-1, were significantly upregulated in M2 macrophages (Fig. 2b), indicating the successful polarization of monocytes. The M2 macrophages were co-cultured with gastric cancer cells (MKN-45, AGS, MGC-803, and HGC-27), breast cancer cells (MDA-MB-231, MDA-MB-435, and MDA-MB-468), or melanoma cells (A375). The results of the Boyden chamber assays revealed that the number of migrated MKN-45, AGS, MDA-MB-231, MDA-MB-435, or MDA-MB-468 cells was significantly increased when these cells were co-cultured with M2 macrophages (Fig. 2c and d), whereas co-culturing MGC-803, HGC-27, or A375 cells with M2 macrophages did not affect the number of the cancer cells that migrated compared with those observed in the treatment medium-only group (Fig. 2c–e). However, co-culturing M1 macrophages with cancer cells did not affect the number of cancer cells that migrated (Fig. 2c–e), suggesting that M2 macrophages play an important role in gastric and breast cancer cell metastasis. To assess whether the effect of M2 macrophages on cancer cell metastasis was due to M2 macrophage secretions, the M2 macrophage culture supernatant was added to cultured cancer cells. The results indicated that the M2 macrophage culture supernatant significantly promoted the migration of gastric cancer cells (MKN-45 and AGS) and breast cancer cells (MDA-MB-231, MDA-MB-435, and MDA-MB-468) (Fig. 2c, d), suggesting that M2 macrophage-derived secretions stimulated the metastasis of these cancer cells. MKN-45 or AGS cells exhibited a stretched and elongated morphology after co-culturing with M2 macrophages or with the M2 culture supernatant, whereas co-cultures with this cell type or the supernatant did not affect the morphology of MGC-803 and HGC-27 cells (Fig. 2f). These results suggested that the morphological changes of MKN-45 and AGS cells facilitated cancer cell metastasis mediated by M2 macrophages or by the M2 culture supernatant.

To further explore the effects of macrophages derived from human samples on cancer cell metastasis, the peripheral blood monocytes of healthy humans were induced to differentiate into M1 and M2 macrophages using LPS and IL-4, respectively. Then, the cancer cells were co-cultured with isolated M1 or M2 macrophages. The results indicated that co-culturing isolated M2 macrophages and gastric cancer cells (MKN-45 and AGS) or breast cancer cells (MDA-MB-231, MDA-MB-435, and MDA-MB-468) led to a significant increase in the number of migrated cancer cells compared with the control (i.e., medium alone) (Fig. 2g). However, M1 cells had no effect on cancer cell migration (Fig. 2g). The data showed that the monocyte-derived M2 macrophages promoted the migration of gastric cancer cells and breast cancer cells.

To assess the effects of isolated M2 macrophages on cancer cell adhesion and invasion, M1 or M2 macrophages were co-cultured with the cancer cells, and the co-cultures were subjected to adhesion and invasion assays. MKN-45 or AGS gastric cancer cell adhesion was significantly enhanced by co-culturing with M2 but not M1 cells (Fig. 2h). The results obtained using MDA-MB-231, MDA-MB-435, and MDA-MB-468 breast cancer cells were similar to those obtained using gastric cancer cells (Fig. 2h). The invasion assays showed that co-culturing isolated M2 macrophages and gastric or breast cancer cells resulted in a significant increase of cancer cell invasion, whereas isolated M1 cells had no effect on cancer cell invasion (Fig. 2i).

Taken together, the above findings indicate that monocyte-derived M2 macrophages promote the metastasis of gastric and breast cancer cells.

### M2 macrophage-secreted CHI3L1 mediates cancer cell metastasis

**Fig 3:**
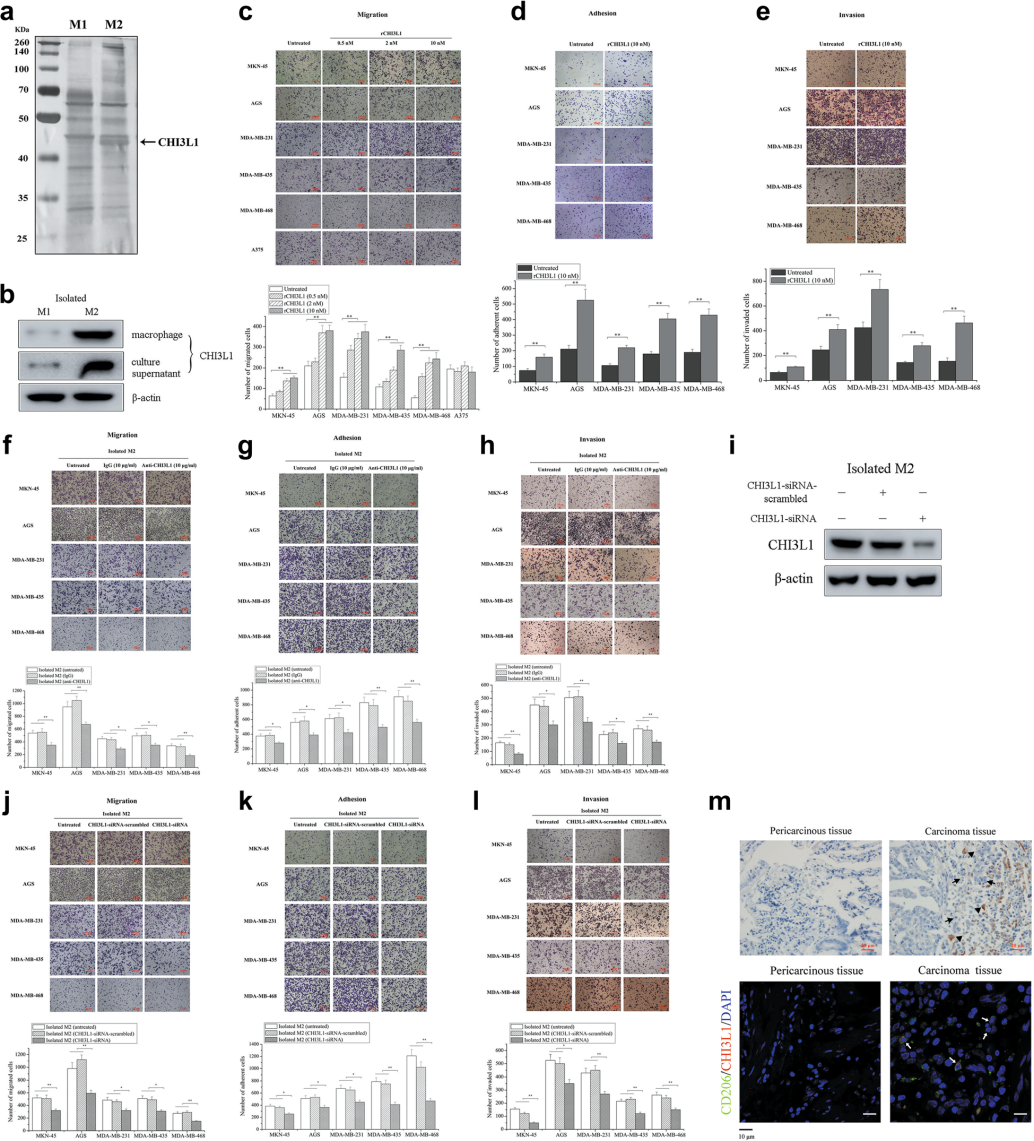
M2 macrophage-secreted CHI3L1 mediates cancer cell metastasis. **a** Screening for different proteins secreted by M1 and M2 macrophages. The secreted proteins were separated using SDS-PAGE and were stained with Coomassie Blue, after which they were identified by mass spectrometry. The *arrow* indicates the protein identified using mass spectrometry. **b** The expression of CHI3L1 in M2 macrophages derived from the peripheral blood monocytes of healthy humans. The peripheral blood monocytes were induced to differentiate into M1 and M2 macrophages. Then monocyte-derived M1 and M2 macrophages or their culture supernatants were subjected to western blotting analysis using anti-CHI3L1 IgG. **c** Effect of recombinant CHI3L1 protein on gastric and breast cancer cell migration in Boyden chamber assays. Gastric cancer cells (MKN-45 and AGS), breast cancer cells (MDA-MB-231, MDA-MB-435, and MDA-MB-468), or melanoma cells (A375) were cultured with the recombinant CHI3L1 protein at different concentrations. 12 h later, the cancer cells were examined by phase-contrast microscopy. *Scalebar*, 100 μm. **d** Roles of recombinant CHI3L1 protein in gastric and breast cancer cell adhesion. Gastric cancer cells (MKN-45 and AGS) or breast cancer cells (MDA-MB-231, MDA-MB-435, and MDA-MB-468) were suspended in serum-free medium and were allowed to adhere to a culture plate coated with fibronectin in the presence of recombinant CHI3L1 protein. After 30 min of incubation, the cells were analyzed using the adhesion assay. *Scalebar*, 100 μm. **e** Effects of recombinant CHI3L1 protein on gastric and breast cancer cell invasion. The invasive capacities of gastric cancer cells (MKN-45 and AGS) or breast cancer cells (MDA-MB-231, MDA-MB-435, and MDA-MB-468) in the presence of the CHI3L1 protein were assessed. The cells were examined after 24 h of treatment. *Scalebar*, 100 μm. **f** The effects of anti-CHI3L1 neutralization on gastric or breast cancer cell migration in Boyden chamber assays. Gastric cancer cells (MKN-45 and AGS) or breast cancer cells (MDA-MB-231, MDA-MB-435, and MDA-MB-468) were co-cultured with the peripheral blood monocytes-derived M2 macrophages of healthy humans in the presence or absence of anti-CHI3L1 antibody; IgG was used as the control. 12 h later, the cancer cells were examined by phase-contrast microscopy. *Scalebar*, 100 μm. **g** The influence of neutralizing CHI3L1 protein on gastric or breast cancer cell adhesion. The adhesion capacity of cancer cells in the presence of the peripheral blood monocytes-derived M2 macrophages and anti-CHI3L1 was evaluated. *Scalebar*, 100 μm. **h** Evaluation of the role of anti-CHI3L1 neutralization in gastric or breast cancer cell invasion. Cancer cells and isolated M2 macrophages were co-cultured and were then subjected to the invasion assay in the presence or absence of the anti-CHI3L1 antibody. *Scalebar*, 100 μm. **i** CHI3L1 gene expression silencing in isolated M2 macrophages from the peripheral blood monocytes of healthy humans. Isolated M2 macrophages were transfected with CHI3L1-siRNA or CHI3L1-siRNA-scrambled. At 24 h after transfection, the CHI3L1 protein level was determined using western blotting. β-actin served as a loading control. **j** Effects of CHI3L1 silencing in isolated M2 macrophages on gastric and breast cancer cell migration. The CHI3L1-silenced isolated M2 macrophages were co-cultured with cancer cells and subjected to cell migration assays. *Scalebar*, 100 μm. **k** The impact of CHI3L1 silencing on gastric and breast cancer cell adhesion. The CHI3L1 gene expression was knocked down in the isolated M2 macrophages. 24 h later, the M2 macrophages were co-cultured with cancer cells, and the adhesion capacities of cancer cells were examined. *Scalebar*, 100 μm. **l** The influence of CHI3L1 silencing on gastric and breast cancer cell invasion. Cancer cells were co-cultured with the CHI3L1-silenced isolated M2 macrophages and then cell invasion assays were conducted. *Scalebar*, 100 μm. **m** Immunohistochemical staining of CHI3L1 protein (*up*) and immunofluorescent double staining of CD206 (*green*) and CHI3L1 (*red*) (*down*) in solid tumors and pericarcinous tissues from patients with gastric cancer. In immunohistochemical staining, the *arrows* indicate cancer cells, and the *triangles* indicate CHI3L1 protein. *Scalebar*, 20 μm. In immunofluorescent double staining, the *arrows* show double-positive macrophages. *Scalebar*, 10 μm. In all the panels, the significant differences are indicated by *asterisks* (*p < 0.05, **p < 0.01)

To determine which proteins secreted by M2 macrophages promote cancer cell metastasis including cancer cell migration, adhesion, and invasion, M1 and M2 macrophage culture supernatants were collected and were resolved on an SDS-PAGE denaturing gel. The results showed that a specific protein band was present in the M2 supernatant but not in the M1 supernatant (Fig. 3a). Mass spectrometry identification showed that this protein was chitinase 3-like protein 1 (CHI3L1, also called YKL-40) (Fig. 3a). To explore the expression of CHI3L1 in M2 macrophages, the peripheral blood monocytes of healthy humans were induced to generate M1 and M2 macrophages, followed by the detection of CHI3L1 protein. Western blot data showed that the CHI3L1 protein was significantly upregulated in the isolated M2 macrophages and their culture supernatants compared with the isolated M1 macrophages (Fig. 3b), suggesting that the CHI3L1 protein might play important roles in promoting cancer cell metastasis.

Recombinant CHI3L1 (rCHI3L1) protein was added to gastric cancer cell culture (MKN-45 and AGS), breast cancer cell culture (MDA-MB-231, MDA-MB-435, and MDA-MB-468), or melanoma cell culture (A375) for cell migration assays using Boyden chambers. The results showed that the migration of gastric or breast cancer cells was significantly enhanced by the recombinant CHI3L1 protein in a dose-dependent manner, whereas CHI3L1 protein had no effect on the migration of A375 melanoma cells compared with the controls (Fig. 3c). To explore the role of CHI3L1 in cancer cell invasion and adhesion, gastric cancer cells (MKN-45 and AGS) or breast cancer cells (MDA-MB-231, MDA-MB-435, and MDA-MB-468) were cultured in media containing the recombinant CHI3L1 protein and were subsequently subjected to invasion and adhesion assays. The results showed that gastric and breast cancer cell adhesion to fibronectin was significantly increased by treatment with CHI3L1 protein (Fig. 3d), indicating that CHI3L1 played a positive role in cancer cell adhesion. Moreover, CHI3L1 protein was shown to significantly enhance the invasiveness of MKN-45, AGS, MDA-MB-231, MDA-MB-435, and MDA-MB-468 cells compared with the controls (Fig. 3e).

To confirm the effects of M2 cells-secreted CHI3L1 protein on cancer cell metastasis (cell migration, adhesion and invasion), a polyclonal anti-CHI3L1 antibody was added to the co-cultures of cancer cells and healthy human peripheral blood monocytes-derived M2 macrophages to neutralize the activity of CHI3L1. The findings revealed that the addition of anti-CHI3L1 led to a significant decrease of gastric and breast cancer cell migration in the presence of isolated M2 macrophages compared with the controls (Fig. 3f), indicating that CHI3L1 played a key role in cancer cell migration. To neutralize the effect of CHI3L1 on gastric and breast cancer cell adhesion, anti-CHI3L1 was added to the cell cultures. In the presence of isolated M2 macrophages, the number of adherent cancer cells was significantly reduced by the antibody compared with the controls (Fig. 3g), showing that CHI3L1 appeared to be required for cancer cell adhesion. When the antibody directed against CHI3L1 (anti-CH3L1) was present in co-cultures of isolated M2 cells and gastric or breast cancer cells, the number of invaded cancer cells was significantly decreased compared with that of untreated co-cultures of isolated M2 cells and cancer cells (Fig. 3h). These findings revealed that monocyte-derived M2 macrophages promoted the metastasis of gastric and breast cancer cells via the CHI3L1 protein.

To explore the effects of silencing CHI3L1 gene expression on cancer cell metastasis, the expression level of the CHI3L1 gene was knocked down using CHI3L1-siRNA in isolated M2 macrophages compared with the control level (Fig. 3i). The results showed that silencing CHI3L1 expression in isolated M2 cells led to significant decreases of cancer cell migration, adhesion, and invasion compared with the control levels (Fig. 3j–l). These findings indicated that CHI3L1 was required for gastric and breast cancer cell metastasis.

To determine the distribution of CHI3L1 protein in cancer tissues, an immunohistochemistry analysis using anti-CHI3L1 and an immunofluorescent double staining using anti-CHI3L1 and anti-CD206 were conducted. The results showed that CHI3L1 protein was localized in the stroma of gastric carcinomas (Fig. 3m). However, no positive signal was observed in the pericarcinous tissue (Fig. 3m). These findings suggested that CHI3L1 protein plays an important role in cancer progression.

Collectively, the data described above indicated that M2 macrophages promote gastric and breast cancer cell metastasis (cell migration, adhesion, and invasion) via the M2 macrophage-secreted CHI3L1 protein.

### The mechanism underlying CHI3L1-activated gastric and breast cancer cell metastasis

**Fig 4:**
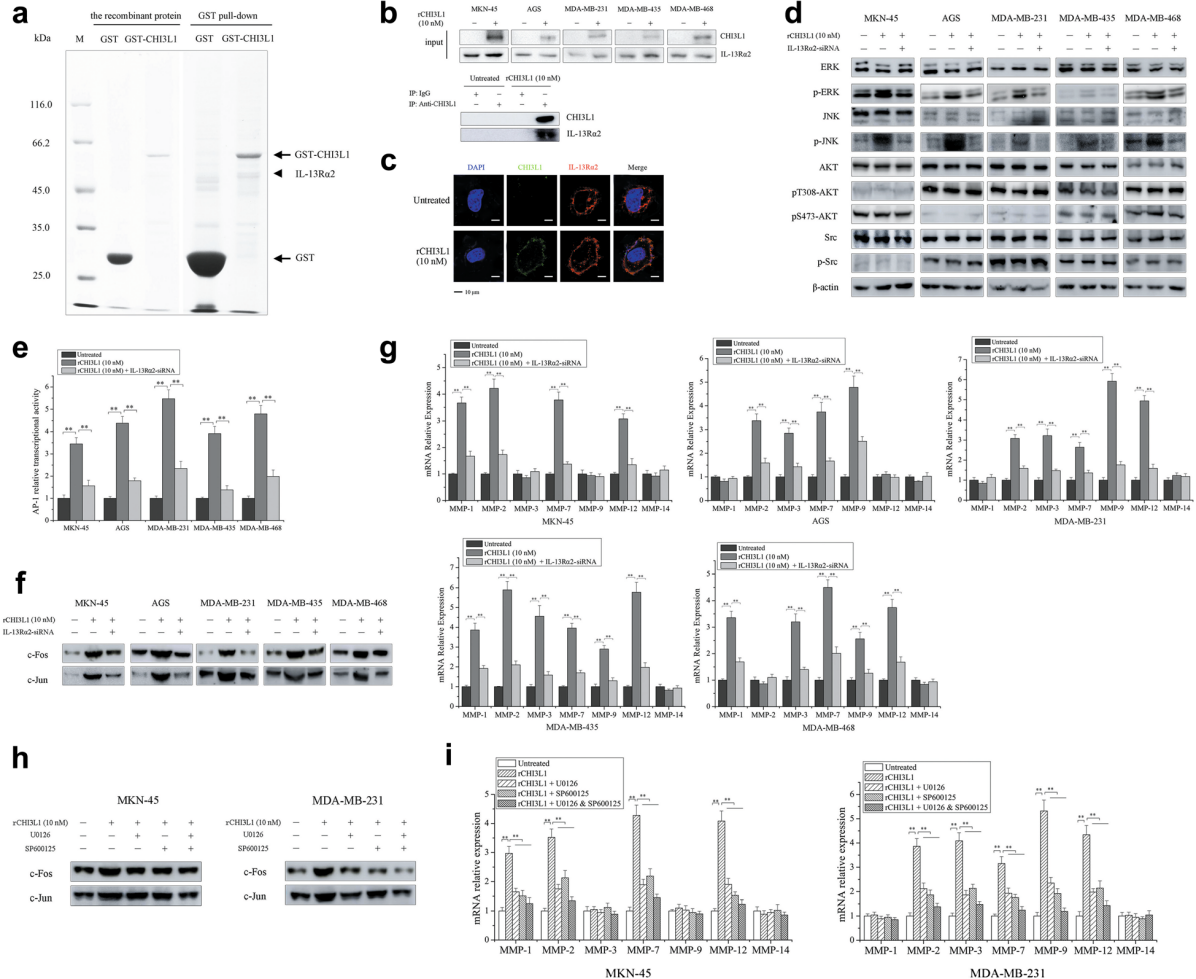
The mechanism underlying the CHI3L1-activated metastasis of gastric and breast cancer cells. **a** Identification of IL-13Rα2 as a CHI3L1-binding membrane receptor. Glutathione-Sepharose beads coupled with GST or GST-CHI3L1 were incubated with isolated gastric and breast cancer cell membrane proteins. The proteins bound to CHI3L1 protein were analyzed by 10% SDS-PAGE with Coomassie staining and were identified by mass spectroscopy. **b** Interaction between CHI3L1 and IL-13Rα2. Gastric and breast cancer cells were incubated with recombinant CHI3L1 (rCHI3L1) for 3 h. Then, the cell membrane proteins were prepared for analysis of the levels of CHI3L1 and IL-13Rα2 using western blot assays (*top*). Additionally, the cell membrane proteins were immunoprecipitated (IP) using either anti-CHI3L1 or an isotype IgG antibody and were analyzed by western blotting (*bottom*). Cells not treated with rCHI3L1 were used as controls. **c** Co-localization of CHI3L1 and IL-13Rα2 proteins on the plasma membrane of cancer cells. MDA-MB-231 cells were incubated with rCHI3L1 for 3 h. Subsequently, the cells were stained with fluorescently labeled anti-CHI3L1 and anti-IL-13Rα2. The nuclei were counterstained with DAPI. The cells were examined using confocal microscopy. **d** Effect of CHI3L1-mediated IL-13Rα2 activation on triggering the activity of the MAPK and PI3K/AKT signaling pathways. Gastric and breast cancer cells with or without IL-13Rα2-siRNA transfection were incubated with rCHI3L1 protein for 3 h. Western blot analysis was conducted to evaluate the levels of total and phosphorylated ERK, JNK, Src, and AKT proteins. β-actin was used as the control. **e** Effects of CHI3L1-mediated IL-13Rα2 activation on AP-1 transcriptional activity in cancer cells. Cancer cells with or without IL-13Rα2-siRNA transfection were transfected with plasmids containing the binding sequence of the AP-1 transcription factor family. Then, recombinant CHI3L1 protein (rCHI3L1) was added to the cell cultures. Dual-luciferase reporter assays were conducted. The level of AP-1 transcriptional activity of the cancer cells was normalized to that of the untreated cells. **f** Influence of CHI3L1-IL-13Rα2 interaction on c-Fos and c-Jun protein expression in the nuclei of cancer cells. Gastric and breast cancer cells with or without IL-13Rα2-siRNA transfection were treated with rCHI3L1 at 10 nM for 3 h. Then, the levels of c-Fos and c-Jun proteins in the cancer cell nuclei were determined by western blotting. **g** Evaluation of MMP expression. Cancer cells with or without IL-13Rα2-siRNA transfection were incubated with rCHI3L1 at 10 nM for 3 h. Subsequently, the cells were subjected to quantitative real-time PCR to determine the levels of MMP expression. **h**, **i** Effects of ERK inhibitor (U0126) and JNK inhibitor (SP600125) on the CHI3L1-induced aggregation of c-Fos and c-Jun protein in the nuclei **h** and MMP gene expression **i** in MKN-45 cells (*left*) and MDA-MB-231 cells (*right*). Prior to the addition of recombinant CHI3L1 protein, cancer cells were treated with U0126 or/and SP600125 at 10 μM for 2 h. Then the cancer cells were incubated with rCHI3L1 protein for 3 h. Significant differences between the treatments are indicated by *asterisks* (*p < 0.05, **p < 0.01)

To reveal the mechanism underlying CHI3L1-activated gastric and breast cancer cell metastasis, cancer cell membrane receptors were screened using GST pull-down assays with recombinant GST-CHI3L1 protein. A specific protein band was observed in the resulting pull-down product (Fig. 4a). Mass spectrometry was used to identify the protein band as interleukin (IL)-13 receptor α2 chain (IL-13Rα2). When cancer cells were incubated with the recombinant CHI3L1 protein (rCHI3L1), the CHI3L1 protein bound IL-13Rα2 (Fig. 4b, bottom panel), indicating an interaction between the two proteins. Moreover, the data revealed that CHI3L1 protein had no effect on IL-13Rα2 expression (Fig. 4b, top panel). Confocal microscopy analysis demonstrated that CHI3L1 protein was co-localized with IL-13Rα2 on the plasma membrane of cancer cells (Fig. 4c). These data indicated that CHI3L1 protein could interact with the IL-13Rα2 receptors on the plasma membrane of MKN-45 and AGS gastric cancer cells and MDA-MB-231, MDA-MB-435, and MDA-MB-468 breast cancer cells. However, the CHI3L1-IL-13Rα2 interaction was not observed in MGC-803 and HGC-27 gastric cancer cells or A375 melanoma cells due to the lack of IL-13Rα2 on their plasma membranes (data not shown). This phenomenon was consistent with the CHI3L1-activated cancer cell metastasis described above (Figs. 2 and 3). In this context, these findings showed that CHI3L1 promoted cancer cell metastasis by interacting with IL-13Rα2 on cancer cell membranes.

Previous studies have revealed that the activation of IL-13Rα2 by IL-13 triggers the MAPK signaling pathway by phosphorylating extracellular signal-regulated kinase (ERK) 1/2 and c-Jun N-terminal kinase (JNK) in pancreatic and ovarian cancers and the phosphoinositide 3-kinase (PI3K)/AKT pathway by phosphorylating Src and AKT in colorectal cancer [17–19]. As shown in Fig. 4d, adding CHI3L1 protein to gastric cancer cell (MKN-45 and AGS) and breast cancer cell (MDA-MB-231, MDA-MB-435, and MDA-MB-468) cultures promoted the phosphorylation of ERK and JNK but not Src and AKT, which was abolished by IL-13Rα2 gene silencing in cancer cells. These results indicated that the activation of IL-13Rα2 by CHI3L1 triggered only the MAPK signaling pathway in gastric and breast cancers.

In the MAPK signaling pathway, the phosphorylation of ERK 1/2 and JNK activates the AP (activator protein)-1 transcription factor family, including c-Fos and c-Jun, and thus promotes the expression of matrix metalloproteinases (MMPs) in cancer cells [19–21]. The luciferase reporter assays showed that AP-1 transcriptional activity in cancer cells was significantly increased by the addition of rCHI3L1 protein compared with the control (Fig. 4e), indicating that AP-1 transcriptional activity was activated by CHI3L1 protein. Western blots showed that the presence of CHI3L1 dramatically elevated the levels of c-Fos and c-Jun proteins in cancer cell nuclei (Fig. 4f), confirming the activation of the AP-1 family by CHI3L1. Further data indicated that the activation of the AP-1 family by CHI3L1 significantly upregulated the levels of MMP expression in cancer cells (Fig. 4g). However, different MMPs were upregulated by CHI3L1 in different cancer cell types. The results showed that knockdown of IL-13Rα2 gene expression in cancer cells significantly reduced the CHI3L1-induced activation of AP-1 transcription factors and upregulation of MMP gene expression in cancer cells (Fig. 4e–g).

To further explore the role of CHI3L1 protein in the CHI3L1-AP-1-MMP pathway, cancer cells were treated with ERK inhibitor (U0126) or JNK inhibitor (SP600125), followed by the addition of CHI3L1 protein in MKN-45 and MDA-MB-231 cells. The data presented that ERK inhibitor or/and JNK inhibitor resulted in decreased aggregation of c-Fos and c-Jun proteins in the nuclei of cancer cells compared with the control (Fig. 4h). At the same time, CHI3L1-induced upregulation of MMP genes in MKN-45 and MDA-MB-231 cancer cells was eliminated when treated with inhibitors (Fig. 4i).

Taken together, these findings demonstrated that the IL-13Rα2 receptor activated the MAPK signaling pathway following CHI3L1 stimulation, thus promoting cancer cell metastasis.

### CHI3L1-mediated promotion of in vivo breast cancer lung metastasis

**Fig 5:**
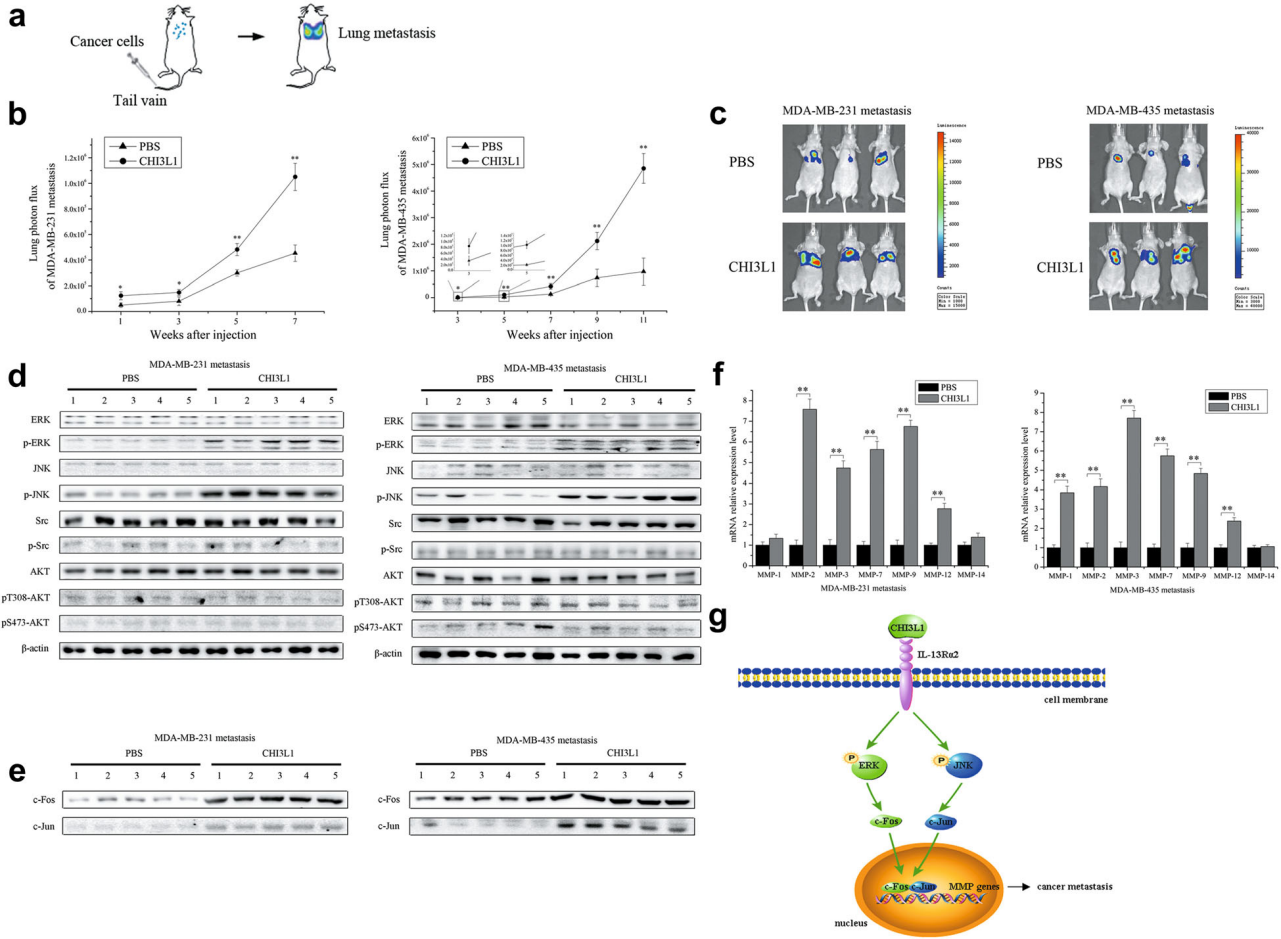
CHI3L1-mediated promotion of in vivo breast cancer lung metastasis. **a** Schematic diagram of cancer cell metastasis to the lung following intravenous injection. **b** Effects of CHI3L1 protein on in vivo breast cancer cell metastasis. MDA-MB-231 and MDA-MB-435 breast cancer cells were intravenously injected into nude mice (*n* = 5/treatment). 3 days later, recombinant CHI3L1 protein was injected into the mice; PBS alone was used as the control. Lung metastasis was monitored via in vivo quantitative luciferase bioluminescence imaging every 2 weeks. The numbers on the horizontal axis indicate the weeks after the injection of CHI3L1 protein. **c** Representative images of MDA-MB-231 cell metastasis (week 7) and MDA-MB-435 cell metastasis (week 11) in mice. **d** Western blotting-based determination of the levels of phosphorylation of ERK, JNK, Src, and AKT proteins in metastatic mouse tumors. The solid tumors separated from mouse lungs were used for this analysis. The treatments are shown at the top. The numbers indicate the number of mice used. β-actin served as the loading control. **e** Levels of c-Fos and c-Jun expression in the nuclei of the metastatic tumor cells of mice treated with CHI3L1 protein or PBS. Western blot analysis was conducted using the solid tumors from mouse lungs. The numbers indicate the number of mice used. **f** MMP gene expression profiles in metastatic mouse tumors. Metastatic mouse tumors treated with CHI3L1 protein or PBS were subjected to quantitative real-time PCR to quantify the gene expression levels. The columns show the data for five mice. Significant differences between the mice treated with CHI3L1 protein and PBS are represented by asterisks (**p < 0.01). **g** Schematic diagram of the signaling cascades activated by CHI3L1 treatment of cancer cells

To investigate the effect of CHI3L1 on breast cancer metastasis in vivo, MDA-MB-231 or MDA-MB-435 cells with a stably integrated luciferase reporter were intravenously injected into nude mice. These mice were then evaluated for cancer metastasis to the lungs (Fig. 5a). The quantitative bioluminescence imaging analysis showed that the metastasis of MDA-MB-231 cells to the lung could be detected 1 week after the injection, whereas MDA-MB-435 cell metastasis was detected 3 weeks after the injection (Fig. 5b). The mice injected with the CHI3L1 protein exhibited much higher lung metastasis luciferase signals than the control (i.e., mice injected with only PBS) (Fig. 5b, c), indicating that CHI3L1 protein promoted cancer metastasis in vivo.

To evaluate whether in vivo cancer metastasis resulted from the activation of the MAPK signaling pathway by the CHI3L1-IL-13Rα2 interaction, the MAPK pathway in the metastatic tumors of mouse lungs was examined. All the mice treated with rCHI3L1 presented remarkably increased levels of phosphorylation of ERK and JNK but not Src and AKT compared with the controls (i.e., mice treated with PBS alone) (Fig. 5d); these results corresponded with those obtained using the cancer cell lines (Fig. 4f). Additionally, the c-Fos and c-Jun expression levels in the nuclei of metastatic tumors of mouse lungs were significantly upregulated in response to rCHI3L1 treatment (Fig. 5e); these results were also consistent with those obtained using the cancer cell lines (Fig. 4h). The MMP gene expression profiles in the metastatic tumors of all the mice treated with rCHI3L1 (Fig. 5f) were similar to those of the cancer cell lines (Fig. 4i). Based on the data obtained in our study, a diagram of the signaling cascades involved in CHI3L1-induced cancer cell metastasis was constructed and is shown in Fig. 5g.

Collectively, these data indicated that CHI3L1 protein promoted in vivo breast cancer metastasis by activating the IL-13Rα2 receptor and subsequently, the MAPK pathway.

### High levels of CHI3L1 protein in the sera of patients with gastric or breast cancer

**Fig 6:**
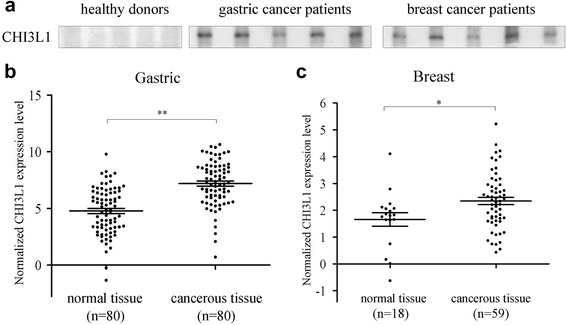
High levels of CHI3L1 protein in the sera of patients with cancer. **a** Detection of CHI3L1 protein in sera. The CHI3L1 protein in the sera of healthy donors and metastatic gastric or breast cancer patients (*n* = 5/group) was immunoprecipitated using anti-CHI3L1. Then, the immunoprecipitated proteins were subjected to western blot analysis using anti-CHI3L1. **b** CHI3L1 expression levels in gastric cancer tissues. CHI3L1 expression levels in the normal and cancerous tissues of the same patients were analyzed using both the Oncomine and the Gene Expression Omnibus (GEO) databases. **c** CHI3L1 expression levels in the normal and cancerous tissues of patients with breast cancer were also evaluated using the Oncomine and GEO databases

To assess whether CHI3L1 protein secreted into the sera of patients with cancer, the levels of CHI3L1 in the sera of healthy donors and metastatic cancer patients were determined. Western blots showed that CHI3L1 protein was present in the sera of gastric and breast cancer patients but not in the sera of healthy donors (Fig. 6a). These data indicated that because CHI3L1 protein could be detected in serum samples, this protein could serve as a marker of metastatic gastric and breast cancer.

Based on examination of the Oncomine and the Gene Expression Omnibus (GEO) databases, high levels of CHI3L1 expression in human tissues correlated with the progression of both gastric cancer (Fig. 6b) and breast cancer (Fig. 6c). These collective data suggested a significant correlation between increased CHI3L1 expression and primary human tumorigenic progression.

## Discussion

Metastasis represents a continuum that begins with cancer cell invasion through the basement membrane into the surrounding stroma, which is followed by extravasation into the circulatory system and the establishment of neoplastic cell colonies at distant sites [22]. There is increasing evidence that macrophages play important roles in modulating the tumor metastatic capacity [23]. Macrophages originate from blood monocytes and differentiate into distinct macrophage types, which are typically identified as M1 (classically activated) and M2 (alternatively activated) macrophages. M1 macrophages are efficient immune effector cells that kill invading microorganisms and tumor cells, present antigens and produce high levels of T-cell stimulatory cytokines. M2 macrophages, however, have poor antigen-presenting capabilities [24]. These cells produce factors that suppress T-cell proliferation and activity and are generally better adapted to promoting angiogenesis and tissue remodeling and repair. Increasing amounts of data have shown that tumor-associated macrophages (TAMs) are polarized M2 macrophages [24]. Genetic studies in mice have indicated that decreased numbers of macrophages in tumors are associated with notably reduced cancer metastasis rates [25]. Although it is known that cytokines secreted by TAMs facilitate cancer cell migration, few TAM cytokines have been investigated. It has been reported that the levels of TAM-derived tumor growth factor-β1 (TGF-β1), epidermal growth factor (EGF), chemokine [C-C motif] ligand 18 (CCL18), interleukin (IL)-18, IL-1β, and IL-8 are correlated with tumor progression [6, 26–30]. Paracrine loops of colony-stimulating factor-1 (CSF-1)/EGF and granulocyte-macrophage colony-stimulating factor (GM-CSF)/CCL18 between carcinoma cells and macrophages have been shown to lead to increased carcinoma cell invasion [31]. However, little is known about the role of the proteins secreted by TAMs in cancer metastasis. The results of this study demonstrated that CHI3L1, which is secreted by M2 macrophages, promoted cancer metastasis by activating the IL-13 receptors of cancer cells. Our study revealed the existence of not only a novel M2-secreted oncogenic protein (not a cytokine) but also a novel mechanism underlying cancer metastasis.

CHI3L1, a glycoprotein, is highly expressed in human tumors [32]. The administration of CHI3L1 has been shown to promote endothelial cell migration and tube formation in vitro but fails to protect cervical cancer cells against apoptosis induced by γ-irradiation [33]. However, the effect of M2 macrophage-derived CHI3L1 on cancer metastasis is not previously addressed. The findings of this study revealed that CHI3L1 specifically bound to the interleukin (IL)-13 receptor α2 chain (IL-13Rα2) of gastric and breast cancer cells, thus promoting cancer metastasis. These findings were consistent with previously reported data [34]. As a binding partner of IL-13Rα2, transmembrane protein 219 (TMEM219) plays a critical role in IL-13Rα2-mediated pulmonary melanoma metastasis [35]. CHI3L1 stimulation leads to TGF-β1 production during lung metastasis [35]. In our study, the results showed that the activation of IL-13Rα2 triggered the downstream MAPK signaling pathway through the phosphorylation of ERK 1/2 and JNK. The phosphorylation of ERK1/2 and JNK led to the recruitment of activator protein (AP)-1 family members (transcription factors) in the nuclei [19, 21] and thus promoted AP-1 target gene expression [36]. The AP-1 target genes are a family of MMPs [19, 36–38]. In the stroma of invasive carcinomas, tumor cells produce a variety of proteases, including various MMPs, to degrade the basement membrane and the surrounding extracellular matrix (ECM), which leads to fibronectin being exposed to the tumor cells [39]. Therefore, CHI3L1 could promote the regional or distal metastasis of cancer. Our data indicated that the CHI3L1 levels in the sera of patients with cancer were significantly elevated compared with those of healthy donors, which suggests its potential as a diagnostic marker of gastric and breast cancers.

## Conclusions

In summary, these data showed that the M2-secreted chitinase 3-like protein 1 (CHI3L1) could promote the metastasis of gastric and breast cancer cells by triggering the mitogen-activated protein kinase (MAPK) signaling pathway. In this context, CHI3L1 has the potential to be a therapeutic target for metastatic cancer. The clinical diagnostic strategy of assessing serum CHI3L1 protein levels merits future investigation.

## Additional file

Additional file 1: Supplemental information. (DOC 75 kb)

## References

[1] Legler DF, Uetz-von Allmen E, Hauser MA (2014). CCR7: Roles in cancer cell dissemination, migration and metastasis formation. Int J Biochem Cell Bio.

[2] Bravo-Cordero JJ, Hodgson L, Condeelis J (2012). Directed cell invasion and migration during metastasis. Curr Opin Cell Bio.

[3] Whiteside T (2008). The tumor microenvironment and its role in promoting tumor growth. Oncogene.

[4] Cho HJ, Jung JI, Lim DY, Kwon GT, Her S (2012). Bone marrow-derived, alternatively activated macrophages enhance solid tumor growth and lung metastasis of mammary carcinoma cells in a Balb/C mouse orthotopic model. Breast Cancer Res.

[5] Kawata M, Koinuma D, Ogami T, Umezawa K, Iwata C (2012). TGF-β-induced epithelial-mesenchymal transition of A549 lung adenocarcinoma cells is enhanced by pro-inflammatory cytokines derived from RAW 264.7 macrophage cells. J Biochem.

[6] Chen J, Yao Y, Gong C, Yu F, Su S (2011). CCL18 from tumor-associated macrophages promotes breast cancer metastasis via PITPNM3. Cancer Cell.

[7] Fantin A, Vieira JM, Gestri G, Denti L, Schwarz Q (2010). Tissue macrophages act as cellular chaperones for vascular anastomosis downstream of VEGF-mediated endothelial tip cell induction. Blood.

[8] Balkwill F (2006). TNF-α in promotion and progression of cancer. Cancer Metast Rev.

[9] Lichtenberger BM, Tan PK, Niederleithner H, Ferrara N, Petzelbauer P (2010). Autocrine VEGF signaling synergizes with EGFR in tumor cells to promote epithelial cancer development. Cell.

[10] Sica A, Mantovani A (2012). Macrophage plasticity and polarization: in vivo veritas. J Clin Invest.

[11] Liu W, Han F, Zhang X (2009). Ran GTPase regulates hemocytic phagocytosis of shrimp by interaction with myosin. J Proteome Res.

[12] Ye T, Tang W, Zhang X (2012). Involvement of Rab6 in the regulation of phagocytosis against virus infection in invertebrates. J Proteome Res.

[13] Chen Y, Jiang C, Jin M, Gong Y, Zhang X (2015). The role of Rab6 GTPase in the maturation of phagosome against *Staphylococcus aureus*. Int J Biochem Cell Bio.

[14] Wu W, Zong R, Xu J, Zhang X (2008). Antiviral phagocytosis is regulated by a novel Rab-dependent complex in shrimp *Penaeus japonicus*. J Proteome Res.

[15] Gong Y, He T, Yang L, Yang G, Chen Y (2015). The role of miR-100 in regulating apoptosis of breast cancer cells. Sci Rep.

[16] Yang G, Gong Y, Wang Q, Wang L, Zhang X. miR-100 antagonism triggers apoptosis by inhibiting ubiquitination-mediated p53 degradation. Oncogene. 2016;1–15 doi:10.1038/onc.2016.270 10.1038/onc.2016.270 27524417

[17] Zhao Z, Wang L, Xu W (2015). IL-13Rα2 mediates PNR-induced migration and metastasis in ERα-negative breast cancer. Oncogene.

[18] Barderas R, Bartolomé RA, Fernandez-Aceñero MJ, Torres S, Casal JI (2012). High expression of IL-13 receptor α2 in colorectal cancer is associated with invasion, liver metastasis, and poor prognosis. Cancer Res.

[19] Fujisawa T, Joshi B, Nakajima A, Puri RK (2009). A novel role of interleukin-13 receptor α2 in pancreatic cancer invasion and metastasis. Cancer Res.

[20] Fujisawa T, Joshi BH, Puri RK (2012). IL‐13 regulates cancer invasion and metastasis through IL-13Rα2 via ERK/AP-1 pathway in mouse model of human ovarian cancer. Int J Cancer.

[21] Kim JH, Kim JH, Kim SC, Yi Y-S, Yang WS (2013). Adenosine dialdehyde suppresses MMP-9-mediated invasion of cancer cells by blocking the Ras/Raf-1/ERK/AP-1 signaling pathway. Biochem Pharmacol.

[22] Tuveson DA, Neoptolemos JP (2012). Understanding metastasis in pancreatic cancer: a call for new clinical approaches. Cell.

[23] Chen Q, Zhang XH-F, Massagué J (2011). Macrophage binding to receptor VCAM-1 transmits survival signals in breast cancer cells that invade the lungs. Cancer Cell.

[24] Qian B-Z, Pollard JW (2010). Macrophage diversity enhances tumor progression and metastasis. Cell.

[25] Yeo E-J, Cassetta L, Qian B-Z, Lewkowich I, Li J-f (2014). Myeloid WNT7b mediates the angiogenic switch and metastasis in breast cancer. Cancer Res.

[26] Hou Z, Falcone DJ, Subbaramaiah K, Dannenberg AJ (2011). Macrophages induce COX-2 expression in breast cancer cells: role of IL-1β autoamplification. Carcinogenesis.

[27] Massague J (2008). TGFβ in cancer. Cell.

[28] Kaler P, Augenlicht L, Klampfer L (2009). Macrophage-derived IL-1β stimulates Wnt signaling and growth of colon cancer cells: a crosstalk interrupted by vitamin D3. Oncogene.

[29] Yang J, Liao D, Chen C, Liu Y, Chuang TH (2013). Tumor-associated macrophages regulate murine breast cancer stem cells through a novel paracrine EGFR/Stat3/Sox-2 signaling pathway. Stem Cells.

[30] Zhong L, Roybal J, Chaerkady R, Zhang W, Choi K (2008). Identification of secreted proteins that mediate cell-cell interactions in an in vitro model of the lung cancer microenvironment. Cancer Res.

[31] Su S, Liu Q, Chen J, Chen J, Chen F (2014). A positive feedback loop between mesenchymal-like cancer cells and macrophages is essential to breast cancer metastasis. Cancer Cell.

[32] Jeet V, Tevz G, Lehman M, Hollier B, Nelson C (2014). Elevated YKL40 is associated with advanced prostate cancer (PCa) and positively regulates invasion and migration of PCa cells. Endocr Relat Cancer.

[33] Ngernyuang N, Francescone RA, Jearanaikoon P, Daduang J, Supoken A (2014). Chitinase 3 like 1 is associated with tumor angiogenesis in cervical cancer. Int J Biochem Cell Bio.

[34] He CH, Lee CG, Dela Cruz CS, Lee CM, Zhou Y (2013). Chitinase 3-like 1 regulates cellular and tissue responses via IL-13 receptor α2. Cell Rep.

[35] Lee C-M, He CH, Nour AM, Zhou Y, Ma B (2016). IL-13Rα2 uses TMEM219 in chitinase 3-like-1-induced signalling and effector responses. Nat Commun.

[36] Kook S-H, Jang Y-S, Lee J-C (2011). Involvement of JNK-AP-1 and ERK-NF-κB signaling in tension-stimulated expression of type I collagen and MMP-1 in human periodontal ligament fibroblasts. J Appl Physiol.

[37] Lin C-M, Chen Y-H, Ma H-P, Wang B-W, Chiu J-H (2012). Silibinin inhibits the invasion of IL-6-stimulated colon cancer cells via selective JNK/AP-1/MMP-2 modulation in vitro. J Agr Food Chem.

[38] Nguyen P, Tsunematsu T, Yanagisawa S, Kudo Y, Miyauchi M (2013). The FGFR1 inhibitor PD173074 induces mesenchymal–epithelial transition through the transcription factor AP-1. Brit J Cancer.

[39] Chiang AC, Massagué J (2008). Molecular basis of metastasis. N Engl J Med.

