# Supplementary material for: Tumor-recruited M2 macrophages promote gastric and breast cancer metastasis via M2 macrophage-secreted CHI3L1 protein
Source: J Hematol Oncol. 2017 Feb 1;10:36. doi: 10.1186/s13045-017-0408-0 (PMC5286803; doi:10.1186/s13045-017-0408-0)
Supplement: Additional file 1: — Supplemental information. (DOC 75 kb) [file 13045_2017_408_MOESM1_ESM.doc]

**Supplemental Information**

**Tumour-recruited M2 macrophages promote cancer metastasis via M2 macrophage-secreted CHI3L1 protein**

Yulei Chen^1^, Siyuan Zhang^1^, Qizhi Wang^2^, Xiaobo Zhang^1^*

*1 College of Life Sciences and Laboratory for Marine Biology and Biotechnology of Qingdao National Laboratory for Marine Science and Technology, Zhejiang University, Hangzhou 310058, The People’s Republic of China*

*2 Department of Gastroenterology, The First Affiliated Hospital of Bengbu Medical College, Bengbu 233030, The People’s Republic of China*

**Supplemental Materials and methods**

**Immunohistochemistry of cancer samples**

The gastric cancer patients were examined by gastroscopy, and gastric cancer tissue specimens were resected. Immunohistochemical staining was performed on 4-μm-thick sections of these specimens. The sections were deparaffinized in xylene and were hydrated using a graded series of alcohol and distilled water. Endogenous horseradish peroxidase (HRP) activity was blocked by incubation in methanol containing 0.3% hydrogen peroxide for 20 min at 25°C. After being washed with distilled water, the sections were autoclaved (121°C) for 5 min. The sections were then incubated with 1% bovine serum albumin (BSA) for 1 h at 25°C and subsequently immunostained using monoclonal mouse anti-HLA-DRα, CD206 (Santa Cruz Biotechnology, Santa Cruz, CA, USA) or polyclonal rabbit anti-CHI3L1 (Abcam, Cambridge, MA, USA) for 1 h at 25°C, after which they were incubated with a biotinylated rabbit anti-mouse IgG secondary antibody (Roche, Basel, Switzerland) for 1 h at 25°C. After incubation with streptavidin-conjugated HRP (Jackson ImmunoResearch, West Grove, PA, USA) for 30 min at 25°C, the sections were treated with 3, 3’-diaminobenzidine tetrahydrochloride (Beyotime Institute of Biotechnology, Shanghai, China). To examine the tumour cells, all the samples were stained with haematoxylin (Beyotime). The specimens were examined by microscopy. Informed consent from the patients and institutional review board approvals were obtained.

**Cell culture**

The gastric cancer cell lines (MKN-45, AGS, MGC-803 and HGC-27), the breast cancer cell lines (MDA-MB-231, MDA-MB-435 and MDA-MB-468), the A375 melanoma cell line and the THP-1 human acute monocytic leukaemia cell line were purchased from The Cell Bank of The Chinese Academy of Sciences (Shanghai, China). The cancer cell lines were maintained in a humidified atmosphere of 5% CO_2_ at 37°C in RPMI 1640 medium (MKN-45 and MGC-803), F-12K Medium (AGS), minimal essential medium (HGC-27) or Dulbecco's Modified Eagle's Medium (A375) supplemented with 10% foetal bovine serum (FBS) (Gibco, Grand Island, NY, USA). The MDA-MB-231, MDA-MB-435 and MDA-MB-468 cell lines were grown at 37°C in a humidified atmosphere (CO_2_ was not present) in Leibovitz's L-15 medium supplemented with 10% FBS (Gibco). All the culture media were purchased from Gibco.

**Polarization of THP-1 monocytes**

THP-1 cells were seeded at 1×10^6^ cells/mL on a culture plate. The cells were treated with 320 nM phorbol 12-myristate 13-acetate (PMA; Sigma, St. Louis, MO, USA) for 6 h. Then, the culture medium was supplemented with either 100 ng/ml lipopolysaccharide (LPS) (Sigma) and 20 ng/ml interferon-γ (PeproTech, Rocky Hill, NJ, USA), for M1 macrophage polarization, or with 20 ng/ml IL-4 (PeproTech) and 20 ng/ml IL-13 (PeproTech), for M2 macrophage polarization. The cells were cultured for an additional 18 h.

**Immunofluorescence assay**

The polarized macrophages were washed three times using phosphate-buffered saline (PBS). Then, the cells were ﬁxed with 4% paraformaldehyde for 10 min and were permeabilized with PBST (PBS, 0.25% Triton X-100) for 10 min at room temperature. After incubation with PBST containing 1% BSA for 30 min at room temperature, the cells were incubated with mouse monoclonal primary antibodies directed against HLA-DRα or CD206 (Santa Cruz Biotechnology) overnight at 4°C;, after which they were incubated with Alexa 594-conjugated donkey anti-mouse IgG (Life Technologies, Carlsbad, CA, USA) for 1 h at room temperature. Subsequently, the cells were counterstained with DAPI (4’, 6-diamidino-2-phenylindole) and were viewed using a confocal laser-scanning microscope (Carl Zeiss, Thornwood, NY, USA).

To evaluate the colocalization of the CHI3L1 and IL-13Rα2 proteins, MDA-MB-231 cells were treated with rCHI3L1 for 3 h and then were washed three times with PBS. Subsequently, the cells were immunofluorescently labelled using anti-CHI3L1 (Abcam) and anti-IL-13Rα2 (R&D Systems, Minneapolis, MN, USA).

**Differentiation of peripheral blood monocytes**

Peripheral blood monocytes derived from healthy human subjects were isolated by density-gradient centrifugation using Histopaque-1077 (Sigma). The isolated monocytes were resuspended in RPMI 1640 medium (Hyclone, Logan, UT, USA) containing 10% FBS (Gibco), plated and allowed to adhere to the culture plates for 12 h. The non-adherent monocytes were then removed. Subsequently, the monocytes were induced to differentiate into M1 macrophages or M2 macrophages by 7 d of treatment with 25 ng/ml lipopolysaccharide (LPS) (Sigma) or 45 ng/ml recombinant human interleukin-4 (IL-4) (PeproTech, Rocky Hill, NJ, USA), respectively (Chen et al, 2011; Day et al, 2013). The differentiation of M1 or M2 macrophages was confirmed by examining their cellular morphology. After being washed three times with PBS, the macrophages were cultured for an additional 2 d. These macrophages were then used for siRNA transfection or co-culturing with cancer cells.

**Boyden chamber cell-migration assay**

Cancer cell migration was evaluated using 24-well Boyden chambers (Corning, Corning, NY, USA) with 8-μm pore-size inserts, as described previously . MKN-45, AGS, MGC-803, HGC-27, MDA-MB-231, MDA-MB-435, MDA-MB-468, or A375 cells suspended in medium lacking FBS were plated onto the inserts in the upper chamber at a density of 3×10^4^ cells/well. Subsequently, medium alone, M1 macrophages, M2 macrophages, the culture supernatant of M2 macrophages or recombinant CHI3L1 protein were added to the lower chamber, allowing the movement of cytokines and proteins but not cells between the upper and lower chambers. The cells were co-cultured at 37°C. After 12 h of co-culturing, the cancer cells on the inserts were fixed with 4% paraformaldehyde (Sigma) and were stained with crystal violet (0.005%) (Beyotime). The number of cells that had migrated were counted using a phase-contrast microscope.

**Adhesion assay**

The abilities of the tested cancer cells to adhere to human fibronectin were determined in 24-well plates. A 0.3-mL aliquot of culture medium containing 10-20 μg/ml of human fibronectin (BD Biosciences, San Jose, CA, USA) was added to each well, which were then incubated at 4°C overnight. The supernatants were then removed, after which the plates were incubated for 30 min at 37°C with culture medium containing 10% BSA (Sigma) (w/v). The wells were then rinsed twice with PBS and were immediately used. Cells that were resuspended in serum-free culture medium were transferred to the coated plates (1×10^5^ cells/well). The cells were allowed to adhere for 30 min at 37°C. Subsequently, the non-adherent cells were removed and the wells were gently washed three times with PBS. The adherent cells were stained using crystal violet (0.005%, Beyotime) and the numbers of cells per field of view were counted under a microscope.

**Invasion assay**

Cancer cell invasion was examined using 24-well invasion chambers with 8-μm pore-size inserts that were coated with Matrigel (Corning). Cancer cells (1.5×10^5^ cells/well) were plated on the inserts and were cultured at 37°C for 24 h. The cells that invaded across the inserts were stained with crystal violet (0.005%, Beyotime) and were counted as cells per field of view under a phase-contrast microscope.

To assess the effect of CHI3L1 neutralization on cancer cell migration, adhesion and invasion, either the rabbit anti-CHI3L1 polyclonal antibody (Abcam) or an isotype-matched IgG control (Beyotime) was added to the culture medium of mcrophages. Then, the cell migration, adhesion and invasion assays were conducted. To assess the influence of CHI3L1-knockdown on cancer cell migration, adhesion and invasion, macrophages were transfected with CHI3L1-siRNA or scrambled siRNA. Twenty hours later, the siRNA-transfected macrophages were cocultured with cancer cells, and the cancer cells were subjected to cell migration, adhesion and invasion assays.

**Quantitative real-time PCR**

SYBR Premix Ex Taq^TM^ (TLi RnaseH plus) (Takara Bio Inc., Shiga, Japan) was used according to the manufacturer’s instructions to quantify the levels of mRNA. The total RNA was extracted from cells or tissues using an RNAprep Pure Cell/Bacteria kit (Tiangen, Shanghai, China). The first-strand cDNA was synthesized from the total RNA using a PrimeScript^TM^ 1st Strand cDNA Synthesis kit (Takara). Then, quantitative real-time PCR was conducted using gene-specific primers to determine the levels of expression of the HLA-DRα, TNFα, IL-6, iNOS, CD204, CD206, CD301, Arginase-1, CHI3L1, MMP (1, 2, 3, 7, 9, 12 and 14) and GAPDH genes. Following initial denaturation at 95°C for 30 s, 50 cycles of PCR amplification were performed at 95°C for 5 s and 60°C for 30 s. Melt curves were generated, and the relative amount of the target gene mRNA was normalized to that of GAPDH.

**Recombinant expression and purification of CHI3L1**

Gene encoding residues 22-383 of human CHI3L1 was subcloned into a pFastBac™ HT B vector (Invitrogen, Carlsbad, CA, USA) using the primers 5’-*GGATCC*TACAAACTGGTCTGCTAC-3’ (BamHI, italics) and 5’-*CTCGAG*CTAC

GTTGCAGCGAGTGCA-3’ (XhoI, italics). Following transformation and amplification in DH10Bac™ *E. coli*, bacmid DNA containing CHI3L1 was transfected into Sf9 insect cells using Cellfectin reagent (Invitrogen) and the baculoviral stock was produced. Protein purification was conducted according to the instructions provided by the manufacturer (GE Healthcare, Waukesha, WI, USA).

**Immunofluorescent double staining of gastric tissues**

Gastric carcinoma tissues and pericarcinous tissues were resected. The samples were deparaffinised, processed for epitope retrieval and stained for CD206 and CHI3L1 protein using monoclonal mouse anti-CD206 (Santa Cruz Biotechnology) and polyclonal rabbit anti-CHI3L1 (Abcam, Cambridge, MA, USA) for 1 h at 25°C. Subsequently the samples were incubated with Alexa Fluor 568-labeled goat anti-rabbit and Alexa Fluor 488-labeled goat anti-mouse (Invitrogen) for 30 min at 25°C. The samples were counterstained with DAPI (Sigma). Fluorescent signals were viewed under a confocal laser-scanning microscope (Carl Zeiss). Informed consent from the patients and institutional review board approvals were obtained.

**Knockdown of CHI3L1 or IL-13Rα2 gene expression**

To silence the expression of the CHI3L1 or IL-13Rα2 gene, the CHI3L1-specific siRNAs (CHI3L1-siRNAs, 5’-GCAACCAGUGGGUAGGAUATT-3’, 5’-GGAGCCA

CAGUCCAUAGAATT-3’ and 5’-GCAUGCUCAACACACUCAATT-3’) and IL-13Rα2-specific siRNAs (IL-13Rα2-siRNAs, 5’-ACCCGACAAUUAUGCUUU GUAGUAA-3’, 5’-CCCGGAUACUUAGGUUAUCUCUAUU-3’) were synthesized by GenePharma (Shanghai, China). Scrambled siRNA (5’-UUCUCCGAACGUGUC

ACGUUU-3’) was used as a negative control. When the macrophages or cancer cells were 30-50% confluent, the cells were transfected with siRNA using Lipofectamine RNAiMAX (Life Technologies) according to the manufacturer’s instructions. The final concentration of siRNA was 100 nM. Twenty-four hours later, the cells were subjected to further experiments.

**Western blot analysis**

Cells or tissues were lysed in RIPA lysis buffer (Beyotime) containing 2 mM phenylmethanesulfonyl fluoride (PMSF) on ice. The proteins in the cell lysates were separated on a 10% SDS-polyacrylamide gel and were then electrotransferred to a nitrocellulose membrane (GE Healthcare) in transferring buffer (25 mM Tris-HCl, 190 mM glycine, 20% methanol). After blocking with 5% non-fat milk in TBST buffer (20 mM Tris-HCl, 150 mM NaCl, 0.05% Tween-20, pH 8.0) for 90 min at room temperature, the membrane was incubated with a primary antibody in TBST buffer containing 1% non-fat milk overnight at 4°C. Subsequently, the membrane was incubated with an HRP-conjugated secondary antibody (BioRad, Hercules, CA, USA) for 2 h at room temperature. The membrane was developed using a Western Lightning Plus-ECL kit (Perkin Elmer, Waltham, MA, USA). The primary antibodies were purchased from Cell Signaling Technology (Danvers, MA, USA), Abcam and R&D Systems.

To determine the levels of the nuclear accumulation of c-Fos and c-Jun, the nuclear proteins of cancer cells or fresh tumour tissues were isolated using a Nuclear and Cytoplasmic Protein Extraction kit (Beyotime) and were then subjected to Western blot analysis.

**Immunoprecipitation**

The cancer cells were grown to 75% confluence and were then incubated with 10 nM rCHI3L1 in serum-free medium for 3 h. Then, the cell membrane proteins were extracted using a ProteoExtract Native Membrane Protein Extraction kit (Calbiochem). These isolates were incubated with anti-CHI3L1 antibody (Abcam) or with the isotype IgG (Beyotime) and with protein A Sepharose (GE Healthcare) in lysis buffer (50 mM Tris-HCl, 150 mM NaCl, 0.5% Triton X-100, pH 7.5) for 1 h at 4°C. After being washed three times with lysis buffer, antibody-conjugated protein A Sepharose was incubated with the extracted membrane proteins overnight at 4°C. Following this incubation, the protein A Sepharose-antibody conjugates were collected by centrifugation at 5,000×*g* for 5 min and were then washed three times using lysis buffer. The immunoprecipitates were analysed by Western blotting using primary antibodies directed against CHI3L1 (Abcam) or IL-13Rα2 (R&D Systems).

**Dual-luciferase reporter assay**

Cancer cells cultured in a 96-well plate at a density of 5×10^4^ cells/well were co-transfected with 0.2 µg of the pAP1-TA-luc (Beyotime) plasmid with 0.02 µg of the control pRL-TK vector (Promega, Madison, WI, USA) using Lipofectamine 2000 reagent (Invitrogen, Carlsbad, CA, USA) according to the manufacturer’s procedures. The pAP1-TA-luc plasmid expressed an AP1 response element (5’-TGACTAATGAC TAATGACTAATGACTAATGACTAATGACTAA-3’) for the binding of AP-1 transcription factor family members . Six hours after transfection, fresh medium was added to the cells and they were cultured for an additional 24 h. These cells were then incubated with the recombinant CHI3L1 protein (rCHI3L1) (10 nM) for 3 h in serum-free medium. For the dual-luciferase reporter assays, the cells were harvested in passive lysis buffer and firefly luciferase and *Renilla* luciferase activities were performed using the Dual-Glo® Luciferase Assay System according the manufacturer’s protocol (Promega). The levels of firefly luciferase activity of pAP1-TA-luc were normalized to that of the control activity of *Renilla* luciferase pRL-TK.

To examine the effects of IL13-Rα2 silencing on AP-1 transcriptional activity, cancer cells were transfected with IL13-Rα2-specific siRNA or scrambled siRNA. Twelve hours later, the cells were transfected with the pAP1-TA-luc and pRL-TK vector.

**Extraction of nuclear proteins**

Nuclear proteins were extracted using a Nuclear and Cytoplasmic Protein Extraction kit (Beyotime) according to the manufacturer’s protocol. Cancer cells were dissolved using 200 μl of cytoplasmic protein extraction reagent A. After vortex-mixing for 5 s, the cells were maintained for 10-15 min on ice. Then, 10 μl of the cytoplasmic protein extraction reagent B was added and the cells were maintained on ice for 1 min. The samples were then centrifuged for 5 min at 12,000-16,000×*g* at 4°C, and the supernatant, consisting of the cytosolic fraction, was removed and was frozen at -80°C. The pellet was resuspended in 50 μl of nuclear protein extraction reagent supplemented with PMSF. After vortex-mixing for 30 min, this mixture was centrifuged for 10 min at 12,000 to 16,000×*g* (4°C),The supernatant containing the nuclear proteins was removed. The protein concentration of the nuclear fraction was determined using a BCA Protein Assay kit (Beyotime).

**Oncomine and GEO analysis**

The levels of CHI3L1 expression in the cancerous and normal tissues of patients with gastric or breast cancers were evaluated using the Oncomine Cancer Microarray database ([http://www.oncomine.org](http://www.oncomine.org/)) and the Gene Expression Omnibus (GEO) database (www.ncbi.nlm.nih.gov/geo/). The Oncomine datasets used were the Cui Gastric dataset for gastric cancer and Curtis Breast dataset for breast cancer. The GEO database used were GSE27342 for gastric cancer and GSE3193 for breast cancer. Significant difference was defined as a *P* value of < 0.05.

**Supplemental References**

1. Cheng GZ, Chan J, Wang Q, Zhang W, Sun CD, et al. Twist transcriptionally up-regulates AKT2 in breast cancer cells leading to increased migration, invasion, and resistance to paclitaxel. Cancer Res. 2007;67**:**1979-1987

2. Watson PA, Chen YF, Balbas MD, Wongvipat J, Socci ND, et al. Constitutively active androgen receptor splice variants expressed in castration-resistant prostate cancer require full-length androgen receptor. P Natl Acad Sci USA. 2010;107**:**16759-16765

3. Fornari F, Gramantieri L, Ferracin M, Veronese A, Sabbioni S, et al. MiR-221 controls CDKN1C/p57 and CDKN1B/p27 expression in human hepatocellular carcinoma. Oncogene. 2008;27**:**5651-5661
